# Bonobos Extract Meaning from Call Sequences

**DOI:** 10.1371/journal.pone.0018786

**Published:** 2011-04-27

**Authors:** Zanna Clay, Klaus Zuberbühler

**Affiliations:** 1 School of Psychology, University of St Andrews, St Andrews, Scotland, United Kingdom; 2 Twycross Zoo, Twycross, Atherstone, United Kingdom; University of Rennes 1, France

## Abstract

Studies on language-trained bonobos have revealed their remarkable abilities in representational and communication tasks. Surprisingly, however, corresponding research into their natural communication has largely been neglected. We address this issue with a first playback study on the natural vocal behaviour of bonobos. Bonobos produce five acoustically distinct call types when finding food, which they regularly mix together into longer call sequences. We found that individual call types were relatively poor indicators of food quality, while context specificity was much greater at the call sequence level. We therefore investigated whether receivers could extract meaning about the quality of food encountered by the caller by integrating across different call sequences. We first trained four captive individuals to find two types of foods, kiwi (preferred) and apples (less preferred) at two different locations. We then conducted naturalistic playback experiments during which we broadcasted sequences of four calls, originally produced by a familiar individual responding to either kiwi or apples. All sequences contained the same number of calls but varied in the composition of call types. Following playbacks, we found that subjects devoted significantly more search effort to the field indicated by the call sequence. Rather than attending to individual calls, bonobos attended to the entire sequences to make inferences about the food encountered by a caller. These results provide the first empirical evidence that bonobos are able to extract information about external events by attending to vocal sequences of other individuals and highlight the importance of call combinations in their natural communication system.

## Introduction

A growing body of research on the communicative behaviour of non-human primates has demonstrated that their vocalisations can convey a considerably rich amount of information that is meaningful to receivers (e.g. [1]). For instance, field experiments with various primate species have shown that acoustically distinct alarm calls can inform listeners about specific types of dangers (e.g. [2]–[5]). In some species, there is evidence that signallers produce strings of acoustically variable calls composed in context-specific ways (e.g. [6]–[8]). For example, black-and-white Colobus monkeys (*Colobus polykomos*, *C. guereza)* produce two types of vocalisations to predators, which are arranged in event-specific sequences that are seemingly meaningful to others [8].

Food discovery is another event type during which some primates produce highly context-specific vocalisations. Since food is often patchily distributed and seasonally dispersed, food calls can provide listeners with a useful means to access foraging patches more effectively, while callers appear to gain mainly social benefits [9]–[10]. The production of food-associated calls is not restricted to primates but found in other mammals and some birds, e.g. *Gallus gallus* [11], although relatively little is still known about the type of information conveyed by the calls. At the simplest level, food calls are a basic physiological response indicating that the caller has found something desirable, as demonstrated by receivers approaching food calls more rapidly than other calls [12]–[13] or by triggering foraging behaviour [14]. In some species, food calls appear to provide more detailed information about the food item itself, such as its quality or divisibility, which can be encoded by changes in call rates [12], [15]–[17] or acoustic structure [18]–[19]. For example, Rhesus monkeys (*Macaca mulatta*) produce up to five different food-associated call types [20], which receivers discriminate based on differences in their referential features, rather than their acoustic properties alone [21].

Among the great apes, chimpanzees (*Pan troglodytes*) produce specific calls when discovering food, the ‘rough grunts’ [22]–[23]. The morphology of this call type co-varies with the caller's personal food preference both in captivity and the wild [24]. In a naturalistic playback experiment, it was demonstrated that acoustic variation in this call influences the foraging decisions of receivers, suggesting that the acoustic structure of this graded signal provides meaningful information to other chimpanzees about the quality of food encountered by the caller [25].

What exactly governs receiver responses, however, is a matter of ongoing debate. For instance it is not clear whether receivers respond directly to the calls' physical features or their referential nature, i.e. the causal relation between calls and contexts [26]–[27]. Similarly, signalling is often said to be non-cooperative with signallers merely producing ‘natural’ information in response to biologically relevant events, while any representational content is largely generated by the listeners [28]. These problems are unsolved because the psychological states experienced by primates during call production and perception are rarely investigated. In one recent study, however, food call production in wild chimpanzees was found to co-vary with the presence and arrival of long-term allies, suggesting these calls may be used as a flexible social strategy [10].

Very little is known about how our other closest relative, the bonobo (*Pan paniscus),* naturally communicates about events in the external world. This is despite the fact that some individuals have been remarkably capable in mastering artificial language systems [29]–[30]. In one recent systematic study, Clay & Zuberbühler [31] demonstrated that bonobos also vocalize upon encountering food, but that there are important differences between the two *Pan* species. Whilst both species produce food ‘grunts’, bonobos additionally give four acoustically distinguishable tonal calls when finding food (barks, peeps, peep-yelps, and yelps), which although lying on a graded scale, can be statistically discriminated from one another [31]. Barks are longest in duration, characterised by a distinctive pointed shape and numerous visible harmonic bands. Whilst peeps are also high pitched, they are temporally shorter than barks (and all other calls), with few harmonic bands and had a flat frequency contour. Yelps and peep-yelps are lower in pitch and although share acoustically similarities, yelps possess a marked downward stroke frequency contour, in contrast to the arched contour of the peep-yelps. In terms of production, peeps and barks are most frequently, but not exclusively, given to preferred foods, while yelps and grunts are more often, but not exclusively, given to less preferred foods. Peep-yelps are produced broadly, although they also tend to occur more to mid- and lesser-preferred foods. In sum, the link between individual call types and perceived food quality is only probabilistic in bonobos. One important consequence of this is that different food calls themselves do not appear to allow listeners to make strong predictions about the type of food encountered by the caller. However, in contrast to chimpanzees, bonobos regularly combine different call types together into longer mixed sequences. The composition of these food call sequences related reliably to food quality, suggesting that listeners can gain information from attending to the call sequences. The hypothesis of meaningful call combinations has been put forward before for bonobos, but has never been tested formally [32]–[33].

In the current study, we examined whether listeners were able to extract meaningful information relating to food quality by attending to the composition of these heterogeneous call sequences. To this end, we conducted naturalistic playback experiments in which subjects heard different types of food all sequences, and their subsequent foraging responses were analysed.

## Results

**Figure 1:**
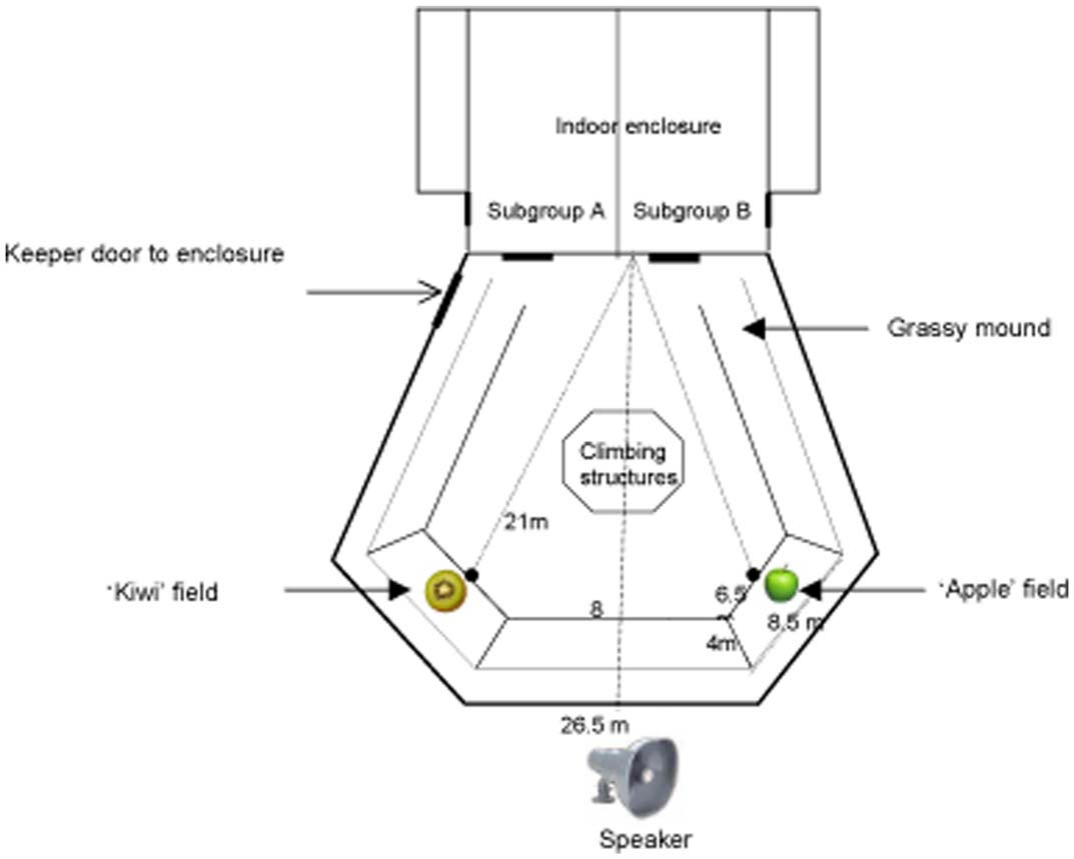
Schematic layout of the bonobo facility at Twycross Zoo, including location of playback equipment and artificial food sites.

The study was conducted at Twycross Zoo, UK, between April and July 2009. Individuals were permanently separated into two subgroups that occupied different indoor facilities but shared the same outdoor area via two separate doors in the morning or afternoon, respectively (fig. 1; table 1). We conducted playback experiments in which we broadcast food-calls of an individual of the morning subgroup responding to either high-quality food (kiwi) or low-quality food (apple) to all members of the afternoon subgroup before releasing them into the outdoor enclosure. Our general aim was to simulate a morning group individual discovering food of high or low quality in the outdoor area shortly before the release of the afternoon subgroup. We also included a control condition in which no food calls were played. We then assessed the foraging behaviours of receivers at previously learned locations for high-quality and low-quality food in the outdoor enclosure (fig. 1).

**Table 1:** Composition of the two bonobo subgroups at Twycross Zoo (UK) in April 2009.

Subgroup A (call producers)				Subgroup B (call receivers)			
Name	ID	Sex	Age	Name	ID	Sex	Age
Kakowet	KT	M	07.06.1980	Diatou	DT	F	21.10.1977
Banya	BY	F	16.02.1990	Kichele	JS	M	02.08.1980
Keke	KK	M	02.01.1994	Jasongo	KH	F	19.04.1989
Maringa	MR	F	05.05.1998	Cheka	CK	F	18.03.1996
Bokela	BK	F	14.10.2003	Luo	LU	M	01.12.2002
				Gemena	GM	F	07.11.2005

### Hearing food-associated calling sequences influenced foraging behaviour

**Figure 2:**
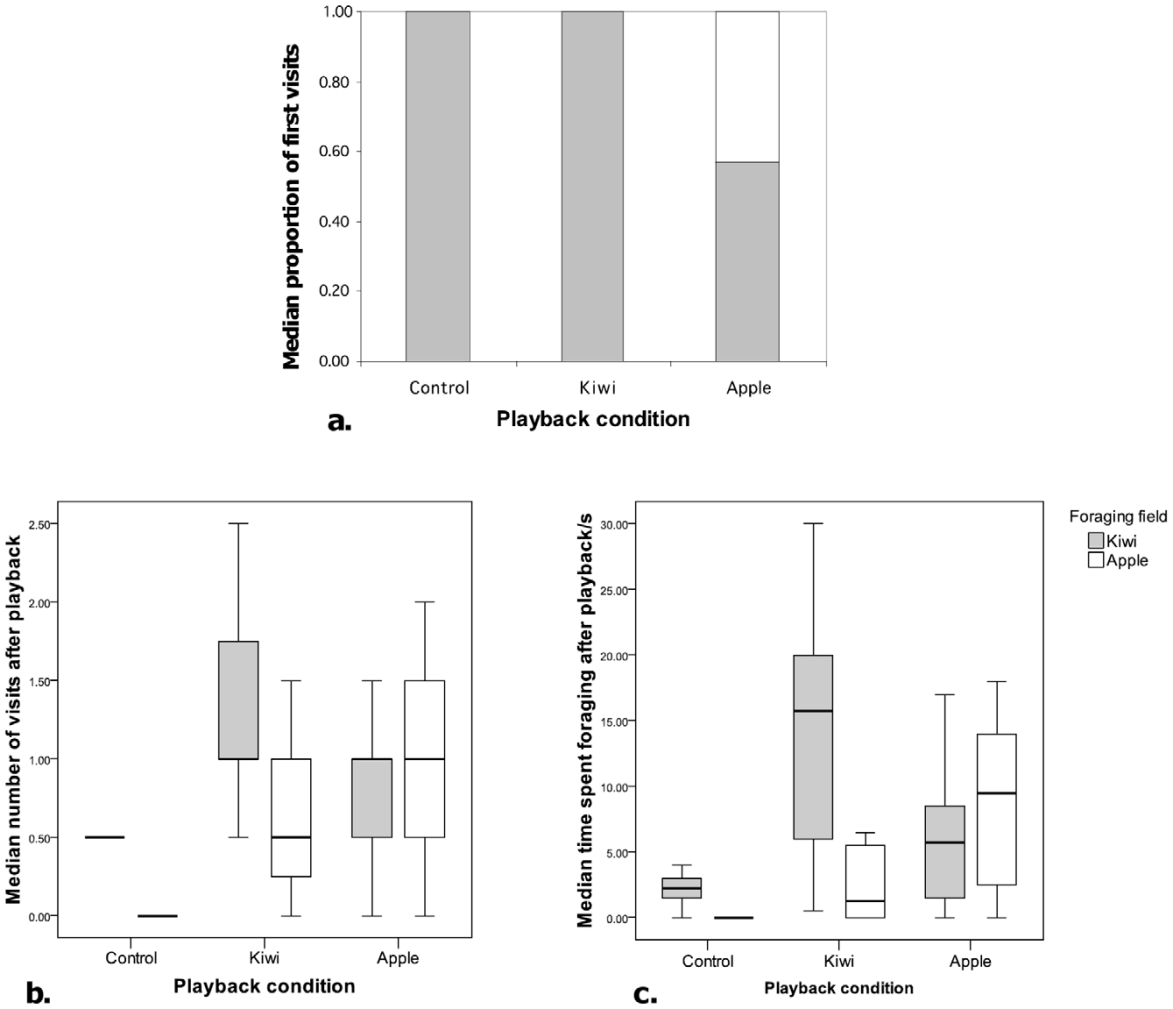
Box plots indicating foraging responses of bonobos (N = 4) following playbacks of food-associated calls given to high value (kiwi) or low value (apple) foods. (a) Site of first entry expressed as a median proportion of the individual's median choices per condition; (b) median number of visits per trial; (c) median time spent foraging following playback (s). Box plots illustrate medians, inter-quartile ranges, and highest and lowest values, excluding outliers.

Following release, there was a strong baseline preference for the highly preferred ‘kiwi’ field. This was particularly evident in the control condition (when no food was presented), in which individuals were more likely to visit the kiwi field first and more often, as well as devoting more foraging effort to it compared to the apple field (fig. 2). Despite this baseline bias, playbacks of food-associated calls had an overall significant effect on the individuals' first choice of fields (χ^2^ (2) = 16.347, p<.001; Pearson chi-square, two-tailed; fig. 2a). Playback of call sequences originally given to kiwi resulted in an increase in first visits to the kiwi field, compared to baseline or apple trials. However, this change failed to reach significance due to a ceiling effect caused by the strong baseline bias for kiwi (First arrivals to kiwi site per individual: median N trials: control condition = 3.0 (50% of trials); kiwi playback condition = 6.0 (86%); apple playback condition = 5.0 (50%), all one-way χ^2^ tests: p>.05). Playback of call sequences originally give to apple resulted in a significant increase in the number of first visits to the apple field, compared to baseline or kiwi trials (First arrivals to apple site, per individual: median N trials: control condition = 0.0; kiwi playback condition = 0.0; apple playback condition = 4.0 (40%); both control and kiwi vs. apple: χ^2^ (1, N = 17) = 13.235, p<.001, with Bonferroni corrected alpha .0169). Hearing food-associated call sequences, in other words, influenced the bonobos' foraging decisions against their pre-existing food preference biases. We conducted this first analysis at the group level because individuals almost always foraged as a cohesive unit and, when released, entered the outside enclosure almost simultaneously (table S1).

Next, we determined whether hearing playbacks influenced the number of visits the group made to the two fields (fig. 2b). Again, we found a significant effect of playback condition on the median number of visits made by the group to both the kiwi field (χ^2^ = 6.486, df = 2, p = .034; two-tailed exact Kruskal-Wallis test) and the apple field (χ^2^ = 10.532, df = 2, p = .002; two-tailed exact Kruskal-Wallis test). Post-hoc, pair-wise comparisons using a Bonferroni correction (corrected alpha = .0169) revealed that individuals visited the ‘kiwi’ field more often after hearing ‘kiwi’ call sequences compared to control trials (median_control_ = 0.5; median_kiwi_ = 1.0; median_apple_ = 1.0; N visits to kiwi field, ‘kiwi’ playback vs. control: Mann-Whitney U = 4.5, p = .015). Conversely, we found that individuals visited the apple field more often after hearing playback of ‘apple’ call sequences compared to the control condition (median_control_ = 0.0; median_kiwi_ = 0.5; median_apple_ = 1.0; N visits to apple field, ‘apple’ playback vs control: U = 3, p = .002).

Finally, hearing playbacks of food-associated call sequences had a significant effect on the foraging time devoted by the group at both the kiwi site (χ^2^ = 6.902, df = 2, p = .026; two-tailed exact Kruskal-Wallis test) and the apple site (χ^2^ = 10.876, df = 2, p = .002; two-tailed exact Kruskal-Wallis test; fig. 2c). Pair-wise comparisons (Bonferroni corrected alpha = .0169) revealed that individuals spent more time at the kiwi location after hearing ‘kiwi’ call sequences compared to control condition (median_control_ = 2.25s; median_kiwi_ = 16.50s; median_apple_ = 5.75s; Kiwi site, ‘kiwi’ playback vs. control: Mann-Whitney U = 5, p = .022,) or hearing ‘apple’ call sequences (‘kiwi’ playback vs. ‘apple’ playback: U = 15.5, p = .058). Likewise, individuals spent more time in the apple field after hearing playbacks of ‘apple’ call sequences compared to control trials (median_control_ = 0.0s; median _apple_ = 9.5s; median_kiwi_ = 1.5; apple field, ‘apple’ playback vs. control: Mann-Whitney U = 6, p = .015). Although there was a trend for spending more time foraging at apple after hearing apple playbacks compared to kiwi playbacks, the result did not reach significance (apple vs. kiwi playbacks: U = 20, p>.05).

All previous analyses were based on non-parametric comparisons at the group level. We selected this analysis strategy to avoid problems with data interdependency and type-two clustering errors [34] at the cost of a substantial reduction in statistical power. Thus, in a second set of analyses we relied on Generalized Linear Mixed Models based on Poisson distributions and log link functions (GLMM), which allowed us to maximise the amount of data available by considering the contributions of individuals. We accounted for individual identity by entering it as a random factor, although it may be argued that combining individuals into one model will not completely eliminate the problem of interdependency, The GLMM procedure is an extension of the more widely known General Linear Model (GLM) and is particularly useful for this study because it relaxes assumptions of normal data distribution and an identity link [35].

This GLMM analysis revealed the same main effects, with a significant interaction between playback type and the number of visits to the two fields (two-tailed GLMM, F (2,178) = 5.037, p = .007; fig. 2b; table S2). Pair-wise comparisons revealed that individuals visited the kiwi field significantly more often after hearing ‘kiwi’ call sequences compared to control trials (p = 0.028). They also visited the apple field significantly more often after hearing ‘apple’ call sequences compared to control and kiwi conditions (p<.001; p = 0.008, respectively).

Finally, hearing playbacks of food-associated call sequences had a significant effect on the foraging time devoted by each of the four individuals in the two fields (two-tailed GLMM: F (2, 178) = 120.772, p<.001; fig. 2c, table S3). Pair-wise comparisons revealed that individuals spent more time at the kiwi location after hearing ‘kiwi’ call sequences than ‘apple’ call sequences or compared to control trials (both p<.001). Likewise, individuals spent more time in the apple field after hearing playbacks of ‘apple’ call sequences compared to ‘kiwi’ call sequences or compared to control trials (both p<.001).

### Foraging errors and integration across the call sequence

**Figure 3:**
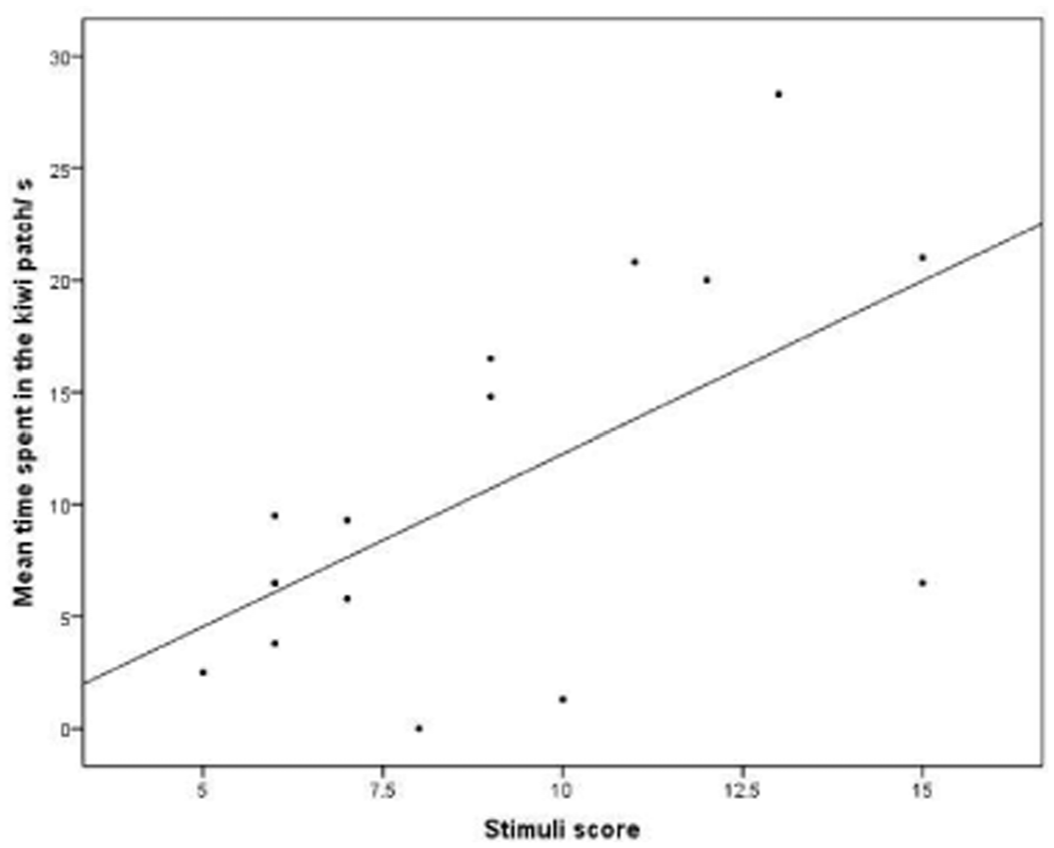
Scatter plot showing the relationship between the food type encountered (highly preferred = kiwi, less preferred = apple) and the cumulative value of the stimuli sequence. Calls were assigned a cardinal score based on how frequently they were produced in response to high vs. low value food (e.g. barks were six times more frequent to high than low preference foods, so that: B = 6.00, P = 1.86, PY = 0.52, Y = 0.12). The relationship between foraging responses and stimuli scores are indicated in table 3.

A key indicator of representationally-based signal processing is that subjects sometimes make mistakes, particularly with signals that are ambiguous or only weakly correlated with specific external events [28]. In our sample, some call sequences were better indicators of high or low food quality than others in terms of call composition, suggesting that if subjects made mistakes then this should happen in response to the more ambiguous sequences (e.g. visiting the apple field after hearing a kiwi sequence). To address this, we assigned a cumulative value to each sequence, based on its call composition. Each call contributed with a value that reflected its association strength with high preference food (table 2). For instance, in natural calling sequences, ‘barks’ were given six times more frequently to high than low preference food (proportion of ‘barks’ in calling sequences to high preference foods = 0.24, vs. low-preference foods = 0.04). Similarly, peeps were given 1.86 times more frequently to high than low preference food (proportion of ‘peeps’ in calling sequences to high preference foods = 0.52 vs. low-preference food = 0.28), and so on. These relative differences resulted in the following cumulative values: B = 6.00, P = 1.86, PY = 0.52, Y = 0.12. We also assigned an ordinal scale, based on the strength of their relationship to high-preference foods, with ‘barks’ being given most frequently to high-preference foods and yelps most infrequently: B = 4, P = 3, PY = 2, Y = 1 (see fig. 3, table 3).

**Table 2:** Relative frequency of food-related call types within natural call sequences given to high and low-value foods by bonobos at Twycross Zoo and the corresponding playback stimuli.

Sequence	Food type			Call type		
		Bark	Peep	Peep-yelp	Yelp	Grunt
**Natural**	High	0.24	0.52	0.22	0.03	0.00
	Low	0.04	0.28	0.42	0.26	0.02
**Playback**	High	0.29	0.50	0.18	0.04	0.00
	Low	0.05	0.13	0.40	0.43	0.00

The values are calculated for the first four calls only; with mean values produced by three individuals in subgroup A (KK, KT, BK) and three other individuals in subgroup B (DT, CK, KH). Results mirror what was previously described from a larger data set from two groups in San Diego (31) showing that although all calls were produced at least once in the high and low contexts, their relative frequency varies with food preference. Barks are produced most typically in response to high value foods; peeps occur often to high value foods, peep-yelps are produced more often to medium-lower value foods, while yelps were typically produced the lower value foods. Grunts were rarely produced in natural call sequences at Twycross, with only 2 individuals producing them, both for low value foods.

**Table 3:** Composition of different call stimuli and resulting behavioural responses in receivers.

Signaller behaviour				Receiver foraging effort (s)		
Food	Sequence	CVO	CVC	Kiwi field	Apple field	Kiwi bias
Kiwi	B B P B	15	19.86	21.0	2.5	9.4
Kiwi	B B P B	15	19.86	6.5	2.5	3.6
Kiwi	B B P PY	13	14.38	28.3	5.0	6.7
Apple	PY B B PY	**12**	**13.05**	**20.0**	**12.0**	**2.7**
Kiwi	P P PY P	11	6.10	79.0	18.8	5.2
Kiwi	PY P P P	11	6.10	20.8	1.8	12.6
Kiwi	P PY PY P	10	4.76	1.3	2.5	**1.5**
Kiwi	P P PY Y	9	4.35	16.5	6.5	3.5
Apple	PY P PY PY	**9**	**3.43**	**14.8**	**3.3**	**5.5**
Apple	Y PY PY P	8	3.02	0.0	15.8	1.0
Apple	Y PY P Y	7	**2.61**	**9.3**	**2.0**	**5.7**
Apple	PY PY Y PY	7	1.69	5.8	20.8	1.3
Apple	Y P Y Y	6	2.20	6.5	14.3	1.5
Apple	Y Y Y P	6	2.20	3.8	40.8	1.1
Apple	PY PY Y Y	6	1.28	9.5	9.0	2.1
Apple	PY Y PY Y	6	1.28	6.5	17.3	1.4
Apple	PY Y Y Y	5	0.87	2.5	10.3	1.2

Receiver foraging effort represents mean time spent foraging at per individual. Cells marked in bold represent 'response errors' where individuals exerted more foraging effort in the incongruent field. Details of how cumulative values were calculated are found in Figure 3. (B = bark, P = peep, PY = peep yelp, Y = yelp). CVO: Cumulative value (ordinal); CVC: Cumulative value (cardinal); Kiwi bias: Relative bias towards the kiwi field.

There was a significant positive correlation between subjects’ foraging effort in the kiwi field and the overall cumulative food value as assessed by the composition of the sequence (time spent: cardinal scale Spearman's rho: N = 17 r_s_ = 0.585, p = .014, fig. 3; ordinal scale: N = 17, r_s_ = 0.575, p = .016). Inspection at the level of individual trials indicated an almost perfect separation of sequences given to apples and kiwis by the cumulative sequence value generated by the constituent calls. One exception was a call sequence given to apples (PY-B-B-PY), which interestingly also triggered almost twice as much searching in the (wrong) kiwi compared to the apple field. Also interesting were two responses to kiwi sequences, which only triggered weak searching in the kiwi field. However, in both cases, search effort in the apple field was also unusually low, suggesting that subjects were generally unmotivated to forage (table 3). In sum, the foraging effort was a strong reflection of the cumulative ‘good food’ score encoded by the sequence.

## Discussion

Human-enculturated bonobos have long been known for their extraordinary representational and communication skills [29]–[30], [36]–[37], but their natural communication behaviour has hardly been investigated. Our study provides progress to this end in showing that bonobos can increase their foraging success by attending to each other's call sequences. Our key finding was that subjects were able to direct their foraging effort to specific locations according to the call sequence presented to them. Whilst we found an unsurprising baseline preference to the high-preference food site, we found that playbacks of high-preference food call sequences resulted in an even greater amount of foraging effort at this site, indicating the calls were meaningful to the receivers. Furthermore, although lack of interest at the low-food preference apple site was to be expected (see baseline trials) we found a significant increase in search effort at this site that only occurred after hearing sequences associated with low-preference food. This result further suggests that individuals incorporated information extracted from the food call sequences to optimise their foraging strategies, in some cases against pre-existing foraging biases.

Although it would be interesting to investigate the mechanism of decision making within the group, we were not able to address this point in our study due to the constraints of working with subjects that were part of a public display (with Zoo regulations prohibiting separation). It is likely that there were individual differences in the ability to assign meaning to the different call sequences used as playback stimuli, either due to individual differences in knowledge or other more immediate factors, such as receiver attention or motivation during the trials. Despite these constraints, we are able to draw the conservative conclusion that at least one individual in our group was able to comprehend the information conveyed by the call sequences, though the results of some trials suggested that several or all group members made their own independent foraging decisions prior to being released into the enclosure. For example, we found that, across trials, different individuals arrived at the food sites first and, in some trials, individuals diverged in their first choices (table S1).

Our results also show that, although chimpanzees and bonobos are phylogenetically closely related, they appear to communicate about food in considerably different ways. In contrast to chimpanzees, who produce an acoustically graded call type that co-varies with food quality [26], bonobos regularly mix several acoustically distinct food call types into heterogeneous strings of vocalisations. Rather than at the level of individual calls, food quality appears related to the probabilistic composition of heterogeneous call sequences [31]. Results from our playback experiment indicate that rather than attending to individual call types, receivers took into account the relative proportions of different calls within the sequence and extracted meaning by integrating information from across the call units.

In addition, the generation of more foraging errors in structurally ambiguous call sequences (which were less strongly indicative of high or low preference foods) indicated that the information extracted from the stimuli sequences was influencing the foraging decisions of the receivers. In a recent discussion, Stegmann [28] argued that in contrast to ‘natural information’, which does not allow for errors, the generation of misrepresentations and errors is a defining feature of what we consider as ‘semantic information’ in animal signals.

Whilst there is a growing body of evidence that numerous monkey species produce strings or sequences of acoustically variable calls composed in context-specific ways, evidence for meaningful signal combinations in apes has been poor (although see [38]). A recent study of gorilla gestural sequences failed to find evidence of syntactic organisation or corresponding semantic content [39]. Results from the current study provide the first empirical evidence that call combinations do play a role in bonobo communication in the foraging context. However, it is important to note that we also did not find any evidence for syntactic rules or that the sequencing structure itself was itself semantically relevant. Thus, although call combinations appear to represent a useful means of communicating information in bonobos, the manner in which bonobos use call combinations contrasts the way linguistic units are combined and structured in human language. This finding highlights the importance of studying non-human primate communication as a means to identify the features of the language faculty that are uniquely human.

One recurrent topic in the animal communication literature is whether signals given in response to external events, such as in this study, should be conceptualised as ‘referential’ or a mere readout of a caller's motivational or internal state [26], [40]–[41]. Great apes, especially chimpanzees and bonobos, are often described as exceedingly ‘emotional’, suggesting that arousal-based explanations may be more in line with the nature of the phenomenon described here (e.g. [26]). In particular, sequences containing a greater amount of calls with presumably high emotional valence may lead receivers to search at the high-value food site. Although we do not discount the integral role motivation plays in animal communication [26], [41], gaining meaningful measurements of internal state or arousal have so far proved very challenging, and thus it has often proved more empirically fruitful to focus on the relation between receiver response and external variables that can be manipulated and measured experimentally [1], [42]. Furthermore, even calls with presumably high motivational content (as may be the case for food discovery) are still able to inform receivers about the external world. This has been demonstrated by studies showing that, regardless of the signaller's motivational state during call production, calls can provide listeners with representational information about external objects and events, in way that can be studied experimentally [1], [27], [41], [43]–[45]. Recent work on the alarm call responses of meerkats (*Suricata suricata*) for example, has demonstrated that both emotional and referential information are encoded into the same signal and develop on different ontogenetic time scales [45]. Meaningful progress will focus more specifically on the motivational experience of the caller and how this influences signal production.

How do receivers extract information from these call sequences? Are they attending to individual calls or do they perceive the sequence as a holistic unit? For example, it could be argued that an increasing number of high-pitched calls, such as peeps and barks, increases the perceived gestalt of the sequence in a discrete way, enabling individuals to make foraging decisions without paying attention to individual calls. Further research will be necessary to address this issue in more detail.

Another related question is whether receivers process vocalisations purely based on their acoustic properties, or whether they attach some semantic value to them. For example, subjects may have learned the contingencies of this particular experiment, e.g. that high-pitched vocalisations were associated with food at one specific location, without any understanding of more general relations between a caller's perceived food quality and the vocal signals produced in response. However, research on food-associated calls of rhesus macaques (*Macaca mulatta*), another primate with a graded vocal system, has demonstrated that individuals categorise calls based on their meaning, not just their acoustic structure alone [31]. Whether bonobos process their own calls in the same way remains open as a topic for future research.

A final unresolved question concerns the function of food call production in bonobos. In capuchins (*Cebus capucinus*), food calls are thought to provide ecological benefits, functioning to announce food ownership and a willingness to defend, thereby resulting in reducing foraging competition from others [46]. In red-bellied tamarins (*Saguinas labiatus*), it has been suggested that food calls are not solely a function of arousal in the presence of highly desirable food patches, but may provide social benefits by attracting allies, even at the cost of increasing feeding competition [9]. A similar effect has recently been indicated in wild male chimpanzees who were found to call more in the presence of close allies and even recommenced calling upon their arrival [10]. For bonobos, it has also been suggested that individuals receive benefits from producing food calls, for instance by attracting mates or potential allies [47]. Further work investigating the interplay and influence of social and ecological variables on the production of food-associated calls in bonobos is required to explore the adaptive significance of these calls in this species.

## Materials and Methods

### Study site and subjects

The study was conducted at Twycross Zoo, UK, between April and July 2009.Individuals were permanently separated into two subgroups that occupied separate indoor facilities but shared the same outdoor area via two separate doors (fig. 1). Subgroup A consisted of 5 individuals (2 adult males and females, 1 juvenile female; range 6–29 years); subgroup B consisted of 6 individuals (1 adult male, 3 adult females, 1 juvenile male, 1 juvenile female; range 4–32 years; table 1). Each subgroup was housed in one of two separated heated indoor halls (62 m^2^) with additional sleeping areas (22 m^2^) and both facilities were separately connected to an outdoor enclosure (588 m^2^). There was no visual contact between indoor and outdoor enclosures, although vocalizations produced outside could be heard indoors. Both subgroups were fed a range of fruits and vegetables twice per day in a scatter feed. Water was freely available. Bonobos were provided with regular enrichment materials and feeds (such as branches, seeds, grapes or frozen juice) as well as supplements such as yoghurt, egg and bread.

### Ethical statement

The Twycross Zoo Ethics and Management Committee and the Zoo Research Coordinator and gave full ethical approval to this behavioural, non-invasive study, which complied with the ethical guidelines set out by the British and Irish Association of Zoos and Aquariums (BIAZA). During all stages of the study, we took steps to ensure that the welfare of all animals was not compromised. No individual showed distress during any part of this study and their participation throughout was voluntary. In order to reduce stress and to comply with Zoo guidelines, we did not separate any individual in any stage of this study.

### Experimental Design

The basic design was to simulate a member of the morning subgroup A finding food shortly before the midday switchover, in order to investigate whether this influenced the subsequent foraging behaviour of subgroup B members. The study consisted of four stages: (1) food preference tests, (2) recording of food-specific calling sequences, (3) establishment of two feeding areas, and (4) playback experiments.

#### 1. Food preference tests

First, we conducted food preference tests to identify a highly valued and a lesser-valued food. Equal sized piles of two foods were placed next to each other on the ground and the first choice was recorded for each individual, repeated across four days, once per day. For each individual, we created a preference matrix based on the percentage of trials in which each food was selected over the other food type across the four sessions. These percentages were then combined to give a cumulative preference score per food, and ranked accordingly (table S4). From eight familiar foods, kiwis and apples were consistently ranked as high and low by all individuals, while both still reliably triggering food calls. We thus selected kiwis and apples as our experimental foods.

#### 2. Recording calls

**Figure 4:**
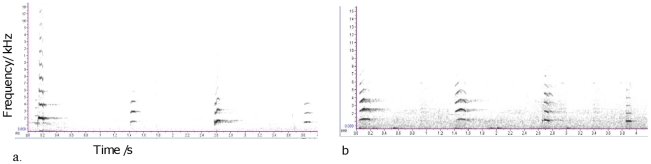
Spectrographic illustrations of two playback stimuli. (a) High value sequence originally given to kiwi consisting of bark/peep/bark/peep and (b) low value sequence originally given to apple consisting of peep-yelp/peep-yelp/yelp/yelp. Recordings of the corresponding call sequences are available as audio S1 and audio S2.

From April to May 2009, we recorded food-associated call sequences given by all individuals feeding in the outdoor enclosure (fig. 4). This allowed us to build up a sound library of call sequences given to kiwi and apples by individuals of the ‘morning’ subgroup for the subsequent playback experiments and to compare their behaviour with a previous study on bonobos [31] (table 2). We recorded vocalizations using a Sennheiser MKH816T directional microphone and a Marantz PMD660 solid-state recorder. Verbal comments were given and later transcribed. We transferred recordings onto a Toshiba Laptop (Equium 1.8 GHz) at a sampling rate of 44.1 kHz with 16-bit accuracy. To control for hunger levels, novelty, and other factors, we only recorded calls produced during first morning feeds. We excluded calls produced by individuals interacting with more than one type of food or when caller identity was uncertain. Calls were recorded from a range of locations from a distance of 2–15 m.

#### 3. Foraging training

During this same period, we established two outdoor foraging patches for the afternoon subgroup in a daily foraging task. Before their midday release, a caretaker hid finely cut pieces (1 cm^2^ pieces, total 300 g) of either apple or kiwi in the grass in one of two 30 m^2^ fields so that they were not visible from a distance. The two fields were on slopes, equidistant to the door (21 m); the distance between them was 8 m. Both field areas were equal (length top = 6.5 m; width = 4.0 m; length bottom = 8.5 m), starting with a flat descent and finishing at the concrete border of the enclosure wall. Kiwi and apple feeds were presented in random order so individuals could not predict which patch was baited so had to inspect the two areas separately. Only one food type was ever provided and no other food or enrichment was provided during training. The keeper always visited both areas, even if no food was placed, to prevent individuals from learning noises associated with scattering food. There were 16 training days for each food type, and 10 control days during which no food was provided. We filmed the individuals' foraging behaviour and kept a daily record of each individual's food encounters (text S1, table S5). Individuals soon learned the two feeding fields and quickly formed a preference for the kiwi field.

#### 4. Playback experiments

In the final step, we conducted playback experiments in which call sequences of members of the morning subgroup were played to individuals in the afternoon subgroup (text S1). The experimental routine was as follows. Around midday, the morning subgroup was brought inside for a seed feed. Live radio broadcasting was played via an inside keeper door to prevent subjects from the morning group from hearing the stimuli (i.e. their own calls) in the subsequent experiment. This was effective as no vocal responses were elicited from any individual during playback trials (except for one apple trial, which was excluded from analysis). Meanwhile, individuals from the afternoon subgroup were waiting to be released through their own door. Beforehand, three key manipulations were carried out. First, a keeper entered the outdoor enclosure from a side door to mimic placing food (none was provisioned). Individuals were familiar with this routine from the previous foraging training. They could not see the event, but could hear the associated sounds. After the keeper's return, subjects heard the opening sounds of the door, which connected the morning subgroup to the outdoor enclosure (to suggest a re-entry of the morning subgroup), although, in reality, no subject was released. A trial was conducted only if (a) no vocalizations had been produced by the morning subgroup for at least 1 minute, (b) individuals of the afternoon subgroup were waiting close (<1–2 m) to the door and not distracted by social activities (play, agonistic, sex) for at least 1 minute; (c) there was no rain or excessive wind outdoors. Communication between a keeper who stayed indoors with the bonobos and the experimenter, who stayed outdoors, was maintained with two-way radios. If these conditions were not met, the trial was either delayed or, in some cases, abandoned. If conditions were met, the afternoon subgroup then heard a 4 s playback of a series of four equally spaced calls extracted from a natural call sequence to either apple or kiwi (to simulate a morning subgroup member finding apple or kiwi) played from their outdoor enclosure (fig. S1). During control trials, all features of the procedure remained the same except that no stimulus was played. One minute after the playback (a sufficient time period for morning subgroup to ‘return’ indoors), the afternoon subgroup was released and their foraging behaviour was monitored for up to 10 min using a camcorder with additional verbal comments. We simultaneously recorded all vocal responses with professional sound recording equipment as previously described. During experimental and control trials, no food was ever provided on either field (to rule out visually-based foraging). To reduce the possibility of extinction, we interspersed a number of refresher days between each trial, i.e. between 1–4 days during which we provided either kiwi or apple pieces on the corresponding fields in random order (N = 28 total).

Zoo regulations prohibited separation of group members (due to stress provoked) so all individuals were released simultaneously into the outdoor enclosure and behavioural response measures were collected while individuals interacted with each other as a group. A total of 28 trials were conducted; three were discarded due to poor weather (preventing the bonobos from being released), one due to unexpected vocalizations (see before), and one due to a communication problem between keeper and experimenter. The remaining 23 trials consisted of N = 10 apple, N = 7 kiwi, and N = 6 control trials, which were completed by four individuals (GM, CK, KH, LU).

The remaining two individuals (DT, JS) were excluded. JS did not complete the training phase and was not motivated to go outdoors due to his low social rank. DT showed no evidence of having learned the food locations during the training phase and showed little interest during the playback phase, only completing 5 of 23 trials, not enough for statistical analyses.

We extracted systematic data on three dependent variables across the different conditions: (a) patch first visited (kiwi vs. apple), (b) time spent actively foraging in each patch (time trespassing, sitting, resting, or sleeping were subtracted), (c) total number of visits per patch (N times entering and exiting the patch areas interrupted by at least one bout of foraging). Because data from individuals were interdependent (we were unable to separate individuals so all individuals foraged together), we conducted our principal analyses at the group level using the median scores for individuals combined per trial. The nature of the data distribution for this method meant that only non-parametric statistics were employed.

Whilst measuring the central tendency of the group across trials reduces the problems of interdependency of the data and type-two clustering errors [34], the cost is a substantial reduction in statistical power. Furthermore, rather than using the foraging behaviours of receivers (upon which our hypothesis is based) as the unit of analysis, the unit of analysis becomes the trials in which the responses of a groups of receivers were measured. We therefore conducted a second analysis using Generalized Linear Mixed Models (Poisson distribution-log link) in order to address the problem of statistical power. The Generalised Linear Mixed Model Procedure (GLMM) is an extension of the General Linear Mixed Model, characterised by a flexible generalization of ordinary least squares regression. The procedure relaxes the assumptions of normal distribution and identity link [35], a crucial prerequisite for the ordinal data of this study. The GLMM procedure enables individual identity to be accounted for (by entering it as a random factor), although this does not completely address the problem of potential inter-dependency in the individuals' foraging decisions.

## Supporting Information

Figure S1 Images depicting (a) the playback speaker positioned during the experimental phase, (b) the view of the sloped outdoor enclosure from the bonobo exit door.
(TIF)

Table S1 **Order that individuals first arrived to one of the two fields per trial, with their choice of field indicated in parentheses (k = kiwi field, a = apple field).** If two or three individuals arrived simultaneously, each of these individuals was given their number plus 0.5 or 0.3, respectively.
(DOC)

Tables S2 **Median number of visits by each individual to the two fields after playbacks of food-associated call sequences.** The top number indicates the median value, with the bottom numbers, indicating the 25 and 75 percentiles. PB  =  Playback condition.
(DOC)

Table S3 **Mean time spent (sec) by each individual at the apple and kiwi slope after hearing food- associated call playbacks.** The top number indicates the median value with the ± indicating the standard errors.
(DOC)

Table S4 **Results of food preference tests conducted on two groups of captive bonobos at Twycross Zoo, UK.**
(DOC)

Table S5 **Direct and indirect experiences by subgroup B individuals during foraging training phase.** Direct experience indicates a foraging event where the individual had physical contact with a food item at the location; indirect experience indicates a foraging event where the individual witnessed another individual eating or on contact with a food item, but themselves did not.
(DOC)

Audio S1 Recording of a call sequence produced by a bonobo in response to finding kiwi, a high-ranked food.
(WAV)

Audio S2 Recording of a call sequence produced by a bonobo in response to finding apples, a low-ranked food.
(WAV)

Text S1 (DOC)
