# Supplementary figures and images for: Bonobos Extract Meaning from Call Sequences
Source: PLoS One. 2011 Apr 27;6(4):e18786. doi: 10.1371/journal.pone.0018786 (PMC3083404; doi:10.1371/journal.pone.0018786)

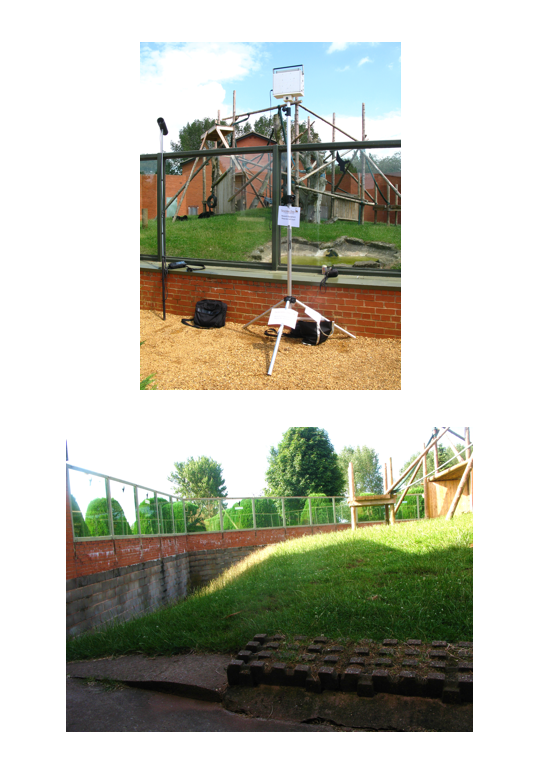

Supplement: Figure S1 — Images depicting (a) the playback speaker positioned during the experimental phase, (b) the view of the sloped outdoor enclosure from the bonobo exit door. (TIF) [file pone.0018786.s001.tif]
