# Supplementary material for: Bonobos Extract Meaning from Call Sequences
Source: PLoS One. 2011 Apr 27;6(4):e18786. doi: 10.1371/journal.pone.0018786 (PMC3083404; doi:10.1371/journal.pone.0018786)
Supplement: Table S1 — Order that individuals first arrived to one of the two fields per trial, with their choice of field indicated in parentheses (k = kiwi field, a = apple field). If two or three individuals arrived simultaneously, each of these individuals was given their number plus 0.5 or 0.3, respectively. (DOC) [file pone.0018786.s002.doc]

**Table S1.**

| **Condition** | **Trial** | Individual* | | | |
| --- | --- | --- | --- | --- | --- |
|  |  | CK | KH | GM | LU |
| Control | 1 | --- | --- | 1 (k) | 2 (k) |
|  | 2 | --- | --- | 1 (k) | 2 (k) |
|  | 3 | 1.5(k) | --- | 1.5 (k) | 3 (k) |
|  | 4 | 2 (k) | 1 (k) | 3 (k) | 4 (a) |
|  | 5 | --- | 2 (k) | 1 (k) | --- |
|  | 6 | --- | 2 (k) | 1 (k) | --- |
| Kiwi | 1 | 3.5 (k) | 3.5 (k) | 1 (k) | 2 (k) |
|  | 2 | 3 (k) | 1 (k) | 2 (k) | 4 (k) |
|  | 3 | 2 (k) | --- | 1 (k) | 3 (k) |
|  | 4 | 2 (k) | 4 (k) | 1 (k) | 3 (k) |
|  | 5 | 1 (k) | 2 (k) | 3 (k) | 4 (k) |
|  | 6 | 3 (k) | 4 (k) | 1 (k) | 2 (a) |
|  | 7 | 1 (k) | --- | 2 (k) | --- |
| Apple | 1 | 2.3 (k) | 2.3 (k) | 2.3 (k) | 1 (k) |
|  | 2 | 2 (k) | 3.5 (a) | 1 (k) | 3.5 (a) |
|  | 3 | 2.5 (k) | 4 (k) | 1 (k) | 2.5 (k) |
|  | 4 | 3 (a) | 2 (a) | 1 (a) | 4 (a) |
|  | 5 | 3 (k) | --- | 1 (a) | 2 (k) |
|  | 6 | 2 (k) | --- | 1 (k) | --- |
|  | 7 | --- | 2 (a) | --- | 1 (a) |
|  | 8 | 1 (k) | 3 (a) | 2 (a) | 4 (a) |
|  | 9 | 2 (a) | 3 (a) | 1 (a) | 4 (a) |
|  | 10 | 2 (k) | 3 (k) | 1 (k) | 4 (a) |

*If several individuals arrived simultaneously they were given an average score. Dashes indicate trials in which a given individual did not enter the site.
