# Supplementary material for: Bonobos Extract Meaning from Call Sequences
Source: PLoS One. 2011 Apr 27;6(4):e18786. doi: 10.1371/journal.pone.0018786 (PMC3083404; doi:10.1371/journal.pone.0018786)
Supplement: Tables S2 — Median number of visits by each individual to the two fields after playbacks of food-associated call sequences. The top number indicates the median value, with the bottom numbers, indicating the 25 and 75 percentiles. PB = Playback condition. (DOC) [file pone.0018786.s003.doc]

**Tables S2.**

| **Individual** | **Kiwi field** | | | **Apple field** | | |
| --- | --- | --- | --- | --- | --- | --- |
|  | Control | Kiwi PB | Apple PB | Control | Kiwi PB | Apple PB |
| GM | 1.00  1, 1 | 2  1, 2 | 1  1, 1.75 | 0.00  0, 0 | 1  0.5, 1 | 2  1, 2 |
| CK | 0  0, 1 | 1  1, 2 | 1  1, 1 | 0  0, 0 | 0  0, 1 | 1  0, 1 |
| LU | 0.50  0, 1 | 1  1, 2 | 0.5  0, 1 | 0  0, 1 | 1  0.5, 1 | 1  1, 1.75 |
| KH | 0  0, 1 | 1  0.5, 2 | 1  0, 1 | 0  0, 0 | 0  0, 0.5 | 1  1, 1.75 |

**PB = playback condition**
