# Supplementary material for: Bonobos Extract Meaning from Call Sequences
Source: PLoS One. 2011 Apr 27;6(4):e18786. doi: 10.1371/journal.pone.0018786 (PMC3083404; doi:10.1371/journal.pone.0018786)
Supplement: Table S3 — Mean time spent (sec) by each individual at the apple and kiwi slope after hearing food- associated call playbacks. The top number indicates the median value with the ± indicating the standard errors. (DOC) [file pone.0018786.s004.doc]

**Table S3.**

| Individual | Kiwi field | | | Apple field | | |
| --- | --- | --- | --- | --- | --- | --- |
|  | Control | Kiwi PB | Apple PB | Control | Kiwi PB | Apple PB |
| GM | 6.33 ±1.33 | 32.86 ±15.00 | 15.60 ±3.75 | 2.17 ±2.17 | 8.28 ±2.91 | 24.90 ±7.25 |
| CK | 0.67 ±0.49 | 17.57 ±5.76 | 6.2 ±2.56 | 0.00 ±0.00 | 1.86 ±0.99 | 8.80 ±3.27 |
| LU | 4.00 ± 1.91 | 25.00 ±9.15 | 5.3 ±1.97 | 1.33 ±0.99 | 5.57 ± 2.03 | 17.20 ±5.85 |
| KH | 1.33 ± 0.84 | 23.57 ±11.73 | 4.3 ±1.71 | 0.00 ±0.00 | 6.86 ±5.15 | 7.20 ±1.66 |
