# Supplementary material for: Bonobos Extract Meaning from Call Sequences
Source: PLoS One. 2011 Apr 27;6(4):e18786. doi: 10.1371/journal.pone.0018786 (PMC3083404; doi:10.1371/journal.pone.0018786)
Supplement: Table S4 — Results of food preference tests conducted on two groups of captive bonobos at Twycross Zoo, UK. (DOC) [file pone.0018786.s005.doc]

**Table S4**.

| Food | Call providers (subgroup A) | | | | | | | | | | | | | | | | | | |
| --- | --- | --- | --- | --- | --- | --- | --- | --- | --- | --- | --- | --- | --- | --- | --- | --- | --- | --- | --- |
|  | KK | |  | | KT | |  | | MR | |  | | BY | |  | | BK | |  |
|  | Rank | | Score | | Rank | | Score | | Rank | | Score | | Rank | | Score | | Rank | | Score |
| Kiwi | 1 | | 65.0 | | 1 | | 67.5 | | 1 | | 67.5 | | 1 | | 70.0 | | 1 | | 70 |
| Banana | 1 | | 65.0 | | 2 | | 62.5 | | 2 | | 62.5 | | 2 | | 60.0 | | 2 | | 60 |
| Orange | 2 | | 45.0 | | 3 | | 50.0 | | 3 | | 47.5 | | 3 | | 47.5 | | 3 | | 45 |
| Apple | 2 | | 45.0 | | 4 | | 40.0 | | 4 | | 42.5 | | 4 | | 42.5 | | 3 | | 45 |
| Food | Call receivers (Subgroup B) | | | | | | | | | | | | | | | | | | |
|  | DT |  | | KH | |  | | CK | |  | | LU | |  | | GM | |  | |
|  | Rank | Score | | Rank | | Score | | Rank | | Score | | Rank | | Score | | Rank | | Score | |
| Kiwi | 1 | 70.0 | | 1 | | 70.0 | | 1 | | 70.0 | | 1 | | 70.0 | | 1 | | 70 | |
| Banana | 2 | 60.0 | | 2 | | 60.0 | | 2 | | 60.0 | | 2 | | 60.0 | | 2 | | 60 | |
| Orange | 3 | 50.0 | | 3 | | 47.5 | | 3 | | 47.5 | | 3 | | 50.0 | | 3 | | 47.5 | |
| Apple | 4 | 40.0 | | 4 | | 42.5 | | 4 | | 42.5 | | 4 | | 40.0 | | 4 | | 42.5 | |

Scores represent the percentage that the given food was chosen other food types
