# Supplementary material for: Bonobos Extract Meaning from Call Sequences
Source: PLoS One. 2011 Apr 27;6(4):e18786. doi: 10.1371/journal.pone.0018786 (PMC3083404; doi:10.1371/journal.pone.0018786)
Supplement: Table S5 — Direct and indirect experiences by subgroup B individuals during foraging training phase. Direct experience indicates a foraging event where the individual had physical contact with a food item at the location; indirect experience indicates a foraging event where the individual witnessed another individual eating or on contact with a food item, but themselves did not. (DOC) [file pone.0018786.s006.doc]

**Table S5.**

| Experience at kiwi or apple site | Individual | | | | | | | | |
| --- | --- | --- | --- | --- | --- | --- | --- | --- | --- |
|  | | GM |  | CK |  | KH |  | LU |  |
|  | | Kiwi | Apple | Kiwi | Apple | Kiwi | Apple | Kiwi | Apple |
| Direct | | 9 | 9 | 9 | 9 | 13 | 14 | 14 | 14 |
| Indirect | | 2 | 3 | 1 | 1 | 1 | 0 | 1 | 0 |
| Total | | 11 | 12 | 10 | 10 | 14 | 14 | 15 | 14 |
