# Supplementary material for: Bonobos Extract Meaning from Call Sequences
Source: PLoS One. 2011 Apr 27;6(4):e18786. doi: 10.1371/journal.pone.0018786 (PMC3083404; doi:10.1371/journal.pone.0018786)
Supplement: Text S1 — (DOC) [file pone.0018786.s009.doc]

# Supporting information

Foraging training experience

We documented the amount of learning experience by counting the number of events in which an individual had (a) direct experience with either fruit patch or (b) indirect experience in which it saw another individual feeding without itself feeding. Four members of the afternoon subgroup (GM, LU, CK, KH) completed the training. The other two (JS, DT) failed to do so, due to social exclusion or lack of motivation. The four successful individuals had direct experiences in at least two thirds of all training days (table S5).

Playback equipment

We used a Nagra DSM speaker/amplifier attached to an Apple Ipod shuffle 2Gb to broadcast the calls. The speaker was positioned equidistant from the two fields on a large tripod 1.85m off the ground so that sounds could be broadcast without interference from the 1.8m glass wall encircling the enclosure. The distance between the speaker and the door entrance was 28m. Although facing the door, the speaker was not visible from the door due to the presence of climbing structures. We began setting up the speaker daily for 10 days before commencing the experiment to allow the bonobos to habituate to its presence. In each trial, the amplitude of the playback stimuli was adjusted so that they sounded natural to an observer experienced with bonobo vocal behaviour. Within this natural range, the stimulus sequence was arbitrarily reset within a 3db range to control for possible amplitude effects. Prior to the experiment, we ensured that the stimuli were audible through the metal door to the individuals indoors.

Stimulus selection

We did not use grunts in this study due to low occurrence and soft amplitude. In order to ensure that the playback stimuli reflected the natural calling behaviour of the bonobos at Twycross Zoo, we compiled the playback stimuli so that they reflected the natural range of sequences produced when encountering high and lower value foods (table 2). We did this by randomly selecting, for each individual, four call sequences to high and low preference foods. For each sequence, we classified the first four calls in the sequence. To cover a range of call sequences naturally produced in response to kiwi and apples, we used a balanced contribution of sequences produced by three individuals (KK, KT, BK) from separate feeding events. We were unable to include calls produced by the other two individuals (MR, BY) due to their low calling rates. If necessary, we edited unequal inter-call intervals to rule out call rate effects.

For some sequences, we also conducted a number of transplantations, whereby we replaced individual calls given to apples with the same call types given to kiwi and vice versa. In doing so, we did not change call types or order (e.g., a ‘peep’ produced as part of a call sequence to kiwi was replaced by a ‘peep’ produced as part of a call sequence to apple). The purpose of this manipulation was to further test our main hypothesis, i.e. that it is the sequence composition, not the acoustic properties of individual calls, that is indicative of the food encountered by a caller. If two recordings from the same individual were comparable in terms of levels of background noise, we modified the sequence composition by transplanting a call produced in one series with a call of the same type from another series, using Adobe Audition. We removed any undesired ambient sounds (e.g. other animals vocalising, motor vehicles, zoo visitors), provided they did not overlap with the stimulus calls. In some cases, it was necessary to reduce the amplitude of the background noise throughout the entire stimulus using a ‘Hann’ band filter. If necessary, the stimulus amplitude was adjusted so that all calls fell within the same amplitude range of 75-80 db. In all cases, amplitude and background noise were only modified if there was no distortion to the overall call sequence so that the sequence continued to sound natural.
